# Two new *Lophoturus* species (Diplopoda, Polyxenida, Lophoproctidae) from Queensland, Australia

**DOI:** 10.3897/zookeys.741.21814

**Published:** 2018-03-07

**Authors:** Cuong Huynh, Anneke A. Veenstra

**Affiliations:** 1 Centre for Cellular and Molecular Biology (CCMB) Deakin University, 221 Burwood Hwy, Burwood, Melbourne, 3125, Australia

**Keywords:** Millipedes, morphological characters, body length, colouration, phylogenetic analysis

## Abstract

*Lophoturus queenslandicus* Verhoeff, 1924 was the first penicillate millipede in the family Lophoproctidae collected from Cairns, a tropical region in Queensland, Australia, to be formally described. Specimens collected from this region in a recent study had the morphological characters known to define this genus. However, their body form and length, as well as dorsal colouration proved to be different, suggesting the possibility of more than one *Lophoturus* species. This assertion was supported by the results of a phylogenetic analysis of DNA extracted and sequenced using 18S and COI regions from *L. queenslandicus* and two undescribed species from this genus. Specimens preserved in ethanol can prove difficult to confidently identify to species level because their colour gradually fades. Examination of live specimens with their body colour visible, together with morphological characters and DNA analysis is the most reliable way of correctly distinguishing between these three species. Two new species, *L. boondallus* **sp. n.** and *L. molloyensis* **sp. n.** collected in Queensland, Australia are described.

## Introduction

Penicillate millipedes from family Lophoproctidae Silvestri, 1897 are characterised as lacking ommatidia, having 13 pairs of legs (except *Lophoturus madecassus* Marquet & Condé, 1950 having only 11 pairs of legs), a gnathochilarium with medial palp only; the 7^th^ and 8^th^ antennal articles are equal in length and reduced sensory cones, coxal glands absent in male, and simple claw structure. These millipedes are commonly found in low light environments such as deep leaf litter or cave habitats. The similarity in their morphological characters proves to be difficult with the classification to genus or species. Ishii et al. (1999) provided a key to the 5 genera in the Lophoproctidae based on the labrum structure and the number of sensilla on the 6^th^ antennal article. These genera are *Alloproctoides* Marquet & Condé, 1950; *Ancistroxenus* Schubart, 1947; *Lophoproctinus* Silvestri, 1948; *Lophoproctus* Pocock, 1894 and *Lophoturus* Brölemann, 1931. Genus *Lophoturus* Brölemann, 1931 is defined by the following characteristics: 0 to 4 pairs of linguiform processes on each side of median cleft of labrum and the 6^th^ antennal article with 3 thick sensilla. There were 27 described species in genus *Lophoturus* worldwide (Nguyen Duy-Jacaquemin and Geoffroy 2003). *L. jianshuiensis* (Ishii & Yin, 2000) from China and two species: *L. speophilus* and *L. humphreysi* (Nguyen Duy-Jacaquemin, 2014) from Christmas Island, Australia were recently added to the species list that totals 30 species to date. *Lophoturus queenslandicus* Verhoeff, 1924 was the first lophoproctid penicillate millipede collected from Cairns, a tropical region in far north Queensland, Australia, formally described (Condé 1979). In this study, penicillate millipedes collected from this region had morphological characters known to define the genus *Lophoturus*. However, their body form and length, as well as dorsal colouration proved to be different, suggesting the possibility of more than one species. The region, where the holotype of *L. queenslandicus* was first found, was visited and fresh specimens collected for comparison with two new *Lophoturus* species collected in Queensland, Australia are described below.

## Materials and methods

**Figure 1.**
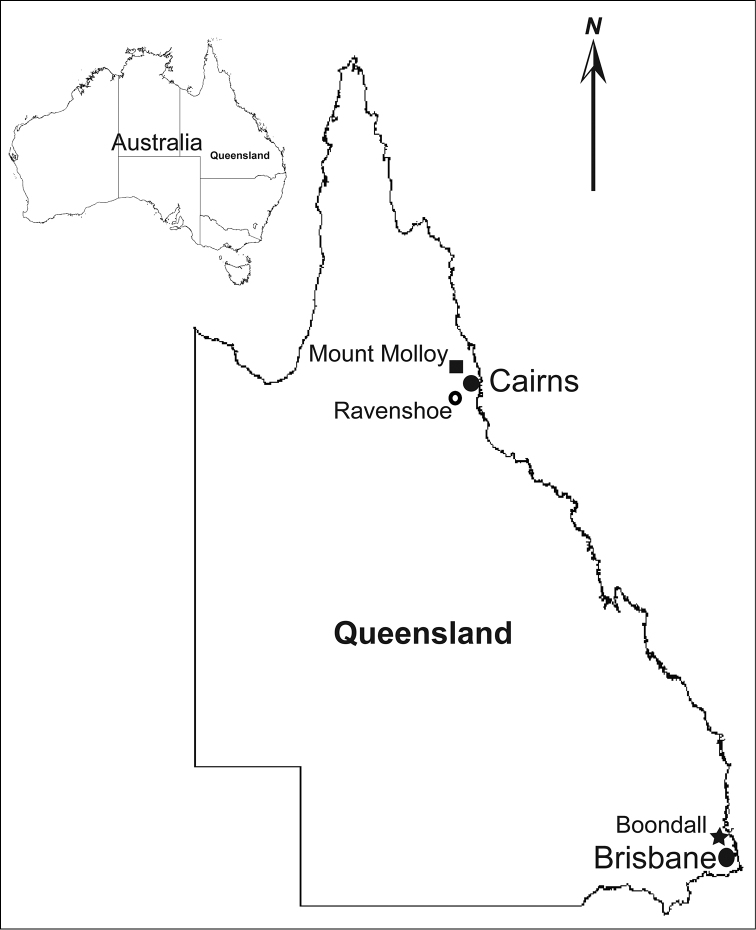
Map of state of Queensland with a map of Australia, indicating type localities of *Lophoturus queenslandicus* Verhoeff, 1924 (⚪) and other two new *Lophoturus* species: *L. boondallus* sp. n. found in Boondall (★), Brisbane and *L. molloyensis* sp. n found in Mount Molloy (⬛), Cairns region, Queensland, Australia. (Not in scale)

*Lophoturus* specimens (Lophoproctidae) were collected from the Cairns region, in tropical far north Queensland and Boondall Wetlands Park in Boondall, a northern suburb of Brisbane, Queensland, Australia (Fig. 1). Specimens of *Lophoturus queenslandicus* were collected from Millstream Falls, Ravenshoe, Tableland region, Queensland for comparison; 17°37'26.56"S, 145°28'42.89"E, elevation 886 m; 15 November 2014 (25 specimens collected: 4 male and 10 female adults, stadium VIII, 13 leg pairs; and 11 subadults with 4 males, 5 females in stadium VII, with 12 leg pairs; and 2 males in stadium VI, with 10 leg pairs). From Lake Eacham, Cairns region, Queensland; 17°16'59.90"S, 145°36'46.92"E, elevation 756 m; 5 December 2013 (eight specimens were collected: 3 males and 5 females (adults). From a roadside of Gillies Highway, 8.5 km SE Goldsborough, Cairns region, Queensland; 17°13'1.98"S, 145°41'55.55"E, elevation 965 m, 6 December 2013 (8 specimens collected: 3 males, 4 females (adults) and one male (12 leg pairs). *Lophoturus boondallus* sp. n. were collected from Boondall Wetlands Park; 27°20'25.85"S, 153° 4'36.94"E, elevation 9 m, 10 November 2015 (12 specimens collected: 2 males and 10 females, all were adult stage). *Lophoturus molloyensis* sp. n. were collected from Mount Molloy (Bakers Road, 3 Km NW from the town of Mount Molloy, along Mulligan Highway), Cairns region, Queensland; 16°41'10.50"S, 145°19'49.43"E, elevation 396 m, 8 December 2016. Seven specimens collected: 2 males and 4 females (adult – stadium VIII), and 1 with 12 leg pairs (subadult – stadium VII).

### Morphometric study

**Light microscopy.** *Lophoturus* specimens were examined and measured using a SMZ 800 stereoscope with an Infinity I camera and an Olympus CX 41 compound microscope with an image capture DP21 digital camera (a reticule with calibration of 0.1 mm stage micrometer and the Infinity I camera program were used for all measurements). Specimens were measured from head to telson, excluding the caudal bundle of trichomes. The sex of the specimens was identified by the presence of reproductive organs on the coxal plates of the 2nd pair of legs.

**Taxonomic drawings** Prepared slides (method described below) were used to complete drawings of the body trichomes using a Nikon drawing tube YID-T attached to a Nikon Eclipse E200 compound microscope.

**Scanning Electron Microscopy** (SEM). Whole specimens were preserved in 80% ethanol and dehydrated by passing through a graded series of ethanol, 80%, 90% and 100%, bathed in acetone for 2 minutes then air dried for a further 2 minutes. Specimens were subsequently mounted on a stub for gold coating using a Fisons sputter coater (0.02 mbar, 18 mA, 2 nm/min), then examined using a JEOL (JSM–IT300 Scanning Electron Microscope). Digital SEM images of the specimens were obtained.

**Morphometric and genetic studies.** The specimen preparation technique of Short and Huynh (2010) was used with modification to permit extraction of DNA for genetic studies. *Lophoturus* specimens were transferred from 80% ethanol onto a slide with a drop of 100% ethanol. Trichomes from the body and caudal bundle were stripped in the presence of ethanol. A resultant slide was then dried for 5 minutes before a drop of DPX was added to mount the slide. This slide was then used to depict the caudal trichomes. Individual stripped specimens were placed in 1.7 mL Eppendorf tubes with the initial extraction buffer solution (Invisorb Spin Forensic Kit (STRATEC Molecular GmbH, D-13125 Berlin, Germany)), left at room temperature for 12 hours then heated for 1 hour at 56 °C before DNA was extracted following manufacturer’s instructions. The extracted DNA was put aside for the genomic DNA study. Cuticles of the same specimens used for DNA extraction were then cleared, dehydrated, stained 1% Fast Green and mounted with DPX mounting medium for microscopic examination following the method of Short and Huynh (2010).

Two common gene markers were used in this study: the small subunit ribosomal RNAs (SSU18S rRNAs) and the mitochondrial cytochrome c oxidase subunit I gene (COI). Both have been used as universal primers for highly conserved gene regions and are common molecular markers used for species detection and identification. The 18S gene marker has been used to elucidate relationships among arthropod groups including crustaceans, insects and myriapods (Turbeville et al. 1991; Luan et al. 2005, Wesener et al. 2010; 2016) This region has also been used to separate penicillate millipede species from genus *Monographis* with similar morphological characters (Huynh and Veenstra 2013; 2015). The COI region was also used because it is used in Barcode of Life (2010–2017) for species identification.

The quality of the DNA extracted from individual specimens of *L. queenslandicus*, *L. boondallus* sp. n. and *L. molloyensis* sp. n. were determined by using a NanoDrop 1000 Spectrophotometer (ND 1000V3.60 software) following manufacturer’s instructions. The primers used for amplification and sequencing of COI were dgLCO1490 and dgHCO2198 (Meyer 2003) obtained from Sigma-Aldrich Co; 18S rDNA were SSUnRNA 1F, SSUnRNA 5R (White et al. 1990) obtained from GeneWorks Pty Ltd. Two microliters of the extracted DNA were sufficient for one Polymerase Chain Reaction (PCR) with 23 µL master mix: 2.5 µL PCR buffer 10x Reaction Buffer plus 15 mM MgCl2, 2.5 µL 25 mM MgCl2, 2.5 µL 2mM dNTP, 2.5 µL BSA (Bovine Serum Albumin, 10% solution), 1 µL forward primer, 1µL reverse primer, 0.1 µL *Taq* (Thermo Fisher Scientific), and 10.9 µL double distilled water. There after the method described by Huynh and Veenstra (2013; 2015) was used.

Representative sequences of *L. queenslandicus*, *L. boondallus* sp. n. and *L. molloyensis* sp. n. were used in a phylogenetic analysis. Partial genomic sequences from these species obtained using the molecular markers SSU18S rRNAs (18S) and the mitochondrial cytochrome c oxidase subunit I gene (COI), were deposited in GenBank: For 18S, the GenBank accession numbers are MG210573 for *L. boondallus* sp. n., MG210574 for *L. molloyensis* sp. n. and MG210575 for *L. queenslandicus*. For COI, the GenBank accession numbers are MG204535 for *L. queenslandicus*; MG204536 for *L. boondallus* sp. n. and MG204537 for *L. molloyensis* sp. n.

18S: The consensus 18S sequences from *L. queenslandicus*, *L. boondallus* sp. n. and *L. molloyensis* sp. n. were used in a BLAST search (http://www.ncbi.nlm.nih.gov) to identify sequences of closely related species. To explore relationships between these species, an entire sequence from *Monographis* sp. collected in China (GenBank accession number AY596371), partial sequences from *M. queenslandicus* (KF147166), *M. dongnaiensis* (KP255446), *Polyxenus lagurus* (EU368619), *Polyxenus fasciculatatus* (AF173235), *Propolyxenus australis* (MF592753), *Unixenus mjobergi* (MF592755), *Lophoproctus coecus* (MF592760), *Chilexenus rosendinus* (MF592765), *Lophoturus madecassus* (MF592767), *Alloproctoides sp.* (MF592759), and two species of pill millipedes (Sphaerotheriida): *Sphaeromimus musicus* (FJ409961) and *Procyliosoma leae* (FJ409955) as an outgroup, were aligned with sequences from *Lophoturus* species using BioEdit (Hall 2010); MEGA7 (Kumar et al. 2016) was used to calculate with maximum composition likelihood method for distance analysis of the nucleotides and a phylogenetic tree was generated using PAUP*4.0b10 (Swofford 2002). A rooted consensus tree of *Lophoturus* species was generated by the bootstrap test with 1000 repetitions.

COI: Sequences of *L. queenslandicus*, *L. boondallus* sp. n. and *L. molloyensis* sp. n. were aligned with the following sequences from related species available on GenBank using a BLAST search (http://www.ncbi.nlm.nih.gov): *Polyxenus lagurus* (HQ966144), *Propolyxenus trivittatus* (MF592724), *Chilexenus rosendinus* (MF592731), *Lophoproctus coecus* (MF592729), *Alloproctoides* sp. (MF592725), *Eudigraphis* sp. (LC010908), a pill millipede *Glomeridella minima* (JN271878) (Sphaerotheriida) and *Pogonsternum* sp. (KU745274) (Polydesmida) as outgroups. Phylogenetic analysis of these species was performed as described above for 18S. The maximum likelihood method was used for pairwise distance analysis of nucleotide composition between these *Lophoturus*.

## Results

### Order Polyxenida Lucas, 1840

#### Family Lophoproctidae Silvestri, 1897

##### Genus Lophoturus

Taxon classificationAnimaliaPolyxenidaLophoproctidae

Brölemann, 1931

###### Type species.

*Lophoturus obscurus* Brölemann, 1931.

*Lophoturus*, is a synonym of *Alloproctinus* Jeekel, 1963 and it was replaced by *Alloproctus* Silvestri, 1948; reassessed by Condé and Nguyen Duy-Jacquemin (1977). It is characterized by 0 to 4 pairs of linguiform processes on each side of median cleft of labrum and antennal article VI with 3 thick sensilla (Ishii et al.1999: 252, key).

##### Lophoturus queenslandicus

Taxon classificationAnimaliaPolyxenidaLophoproctidae

Verhoeff, 1924 (Condé 1979)

###### Note.

*Lophoturus queenslandicus* Verhoeff, 1924 was the first lophoproctid penicillate millipede collected from Ravenshoe, Australia (Condé 1979). Ravenshoe is a town in north-east Queensland previously known as Cedar Creek, where the type specimen of *L. queenslandicus* was collected.

*L. queenslandicus* has 13 pairs of legs, 10 segments and a telson; 9 pleural projections; body covered with barbate trichomes; tergal trichomes form 2 latero-posterior groups with a few trichomes extending anteriorly and these groups are separated by a gap; Chaetotaxy with pubescent oval setae; simple claw; the ornamental trichomes with 8*a*, 1*b* and 2*c* (*c*1 and *c*3); labrum with setose surface and 0 to 1 pairs of linguiform processes; the 6^th^ antennal article with 3 thick sensilla (Condé 1979).

##### Lophoturus boondallus sp. n.

Taxon classificationAnimaliaPolyxenidaLophoproctidae

http://zoobank.org/9FF56672-4164-42F2-84E2-37721098D16E

###### Holotype.

Adult male, stadium VIII – 13 leg pairs stage, collected from Boondall Wetlands Park, Boondall, Brisbane, Queensland; 27°20'25.85"S, 153°4'36.94"E, elevation 9 m. The species was collected from leaf litter near the main entrance of the park on 10 November 2015 by author (CH).

###### Paratypes.

One male and 9 females were collected in the same location and date as holotype. (1 male and 2 females were used for SEM imaging in this study).

###### Etymology.

The species is named *Lophoturus boondallus* sp. n. as they were first found in Boondall Wetlands Park, Boondall, Queensland, Australia.

###### Diagnosis.

*L. boondallus* has the following morphological characteristics: 3 sensilla on the 6^th^ antennal article and labrum with two linguiform processes. These features are typical characteristics of *Lophoturus*. Live specimens from this species are light orange in colour with a round arc-shaped body form in cross section. Adults body length range from 1.6–2.2 mm.

###### Description.

*Measurements*: Holotype male body length 1.9 mm, females (paratypes) (*n* = 8) range from 1.9–2.2 mm. Caudal bundle of the male is slightly narrower in width with 0.6 mm in length than that of the female (0.5 mm) (Fig. 6A–B)

**Figure 2.**
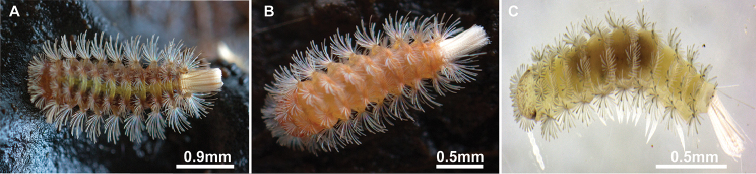
Three *Lophoturus* species were found in state of Queensland, Australia. **A** *L. queenslandicus* Verhoeff, 1924 **B** *L. boondallus* sp. n. and **C** *L. molloyensis* sp. n. These *Lophoturus* species showed differences in body lengths and colour.

***Colouration*.** Head light orange and dark reddish-brown laterally; body light orange, contrasting with their white pleural trichomes and lighter coloured caudal bundle (Figure 2B).

**Figure 3.**
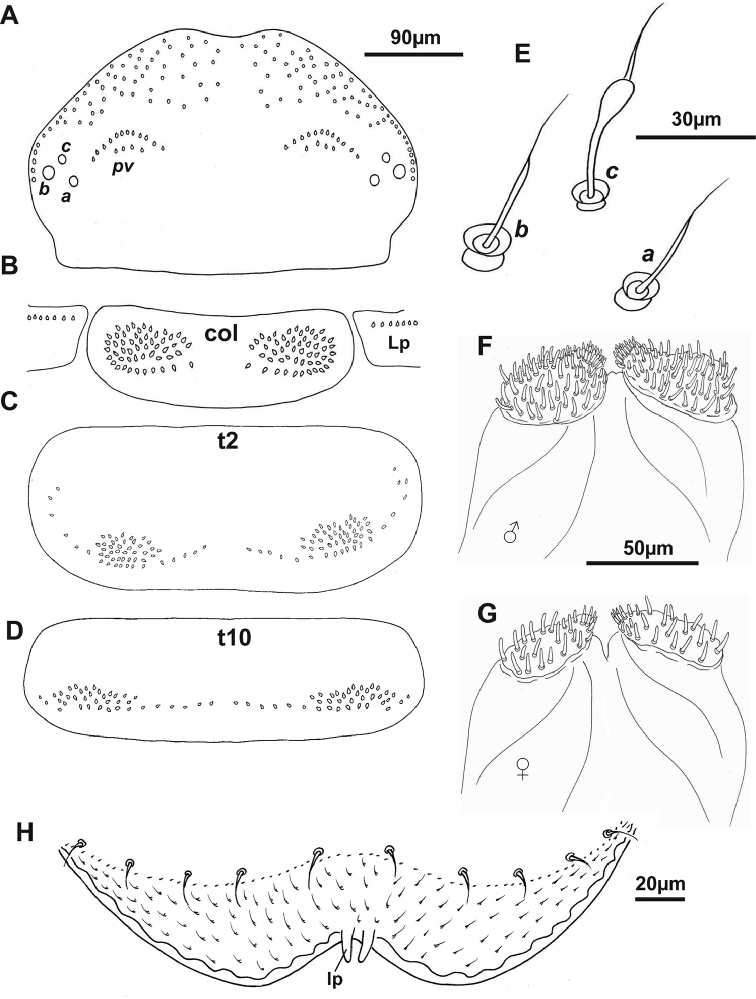
The depiction of holotype of *Lophoturus boondallus* sp. n. **A** A head capsule showed the posterior vertex trichome sockets (**pv**) and trichobothria **B, C, D** Collum (**col**) with the lateral protuberances (**Lp**), tergite 2 (**t2**) and tergite 10 (**t10**), with trichome socket patterns **E** Trichobothria: Trichobothrium ***a*** (located posteriorly with medium socket), trichobothrium ***b*** (located laterally with largest socket) are typical thin sensory hairs and trichobothrium ***c*** with a claviform funicle (located anteriorly with smallest socket) **F** Gnathochilaria of male and **G** Gnathochilaria from female (paratype) **H** Labrum displayed two linguiform processes (**lp**) and setose surface.

***Head*.** Ommatidia absent. Vertex with two posterior trichome groups, a large gap presents between them. Each group consists of 2 rows, the anterior, oblique row has similar sized trichome sockets. Posterior row has fewer trichome sockets with a narrow space between the anterior and posterior rows. Holotype male has 12 + 12 trichome sockets in anterior rows and 4 + 4 trichome sockets in posterior rows (Fig. 3A); paratypes indicate that variation is common in this species, ranging from 12–15 (anterior rows) and 3–7 (posterior rows) (Fig. 6C). Trichobothria: trichobothrium ***a***, located in posterior position with a medium socket; trichobothrium ***b***, lateral position with largest socket; and trichobothrium ***c***, anterior position with smallest socket. Trichobothria ***a*** and ***b*** have typically thin sensory hairs with narrow cylindrical funicles compared to trichobothrium ***c***, with a claviform funicle. Trichobothrium sockets (***a***, ***b*** and ***c***) arranged unevenly between ***ab*** and ***bc***, as trichobothria ***a*** and ***c*** located more inward (Figs 3E, 6D).

**Figure 4.**
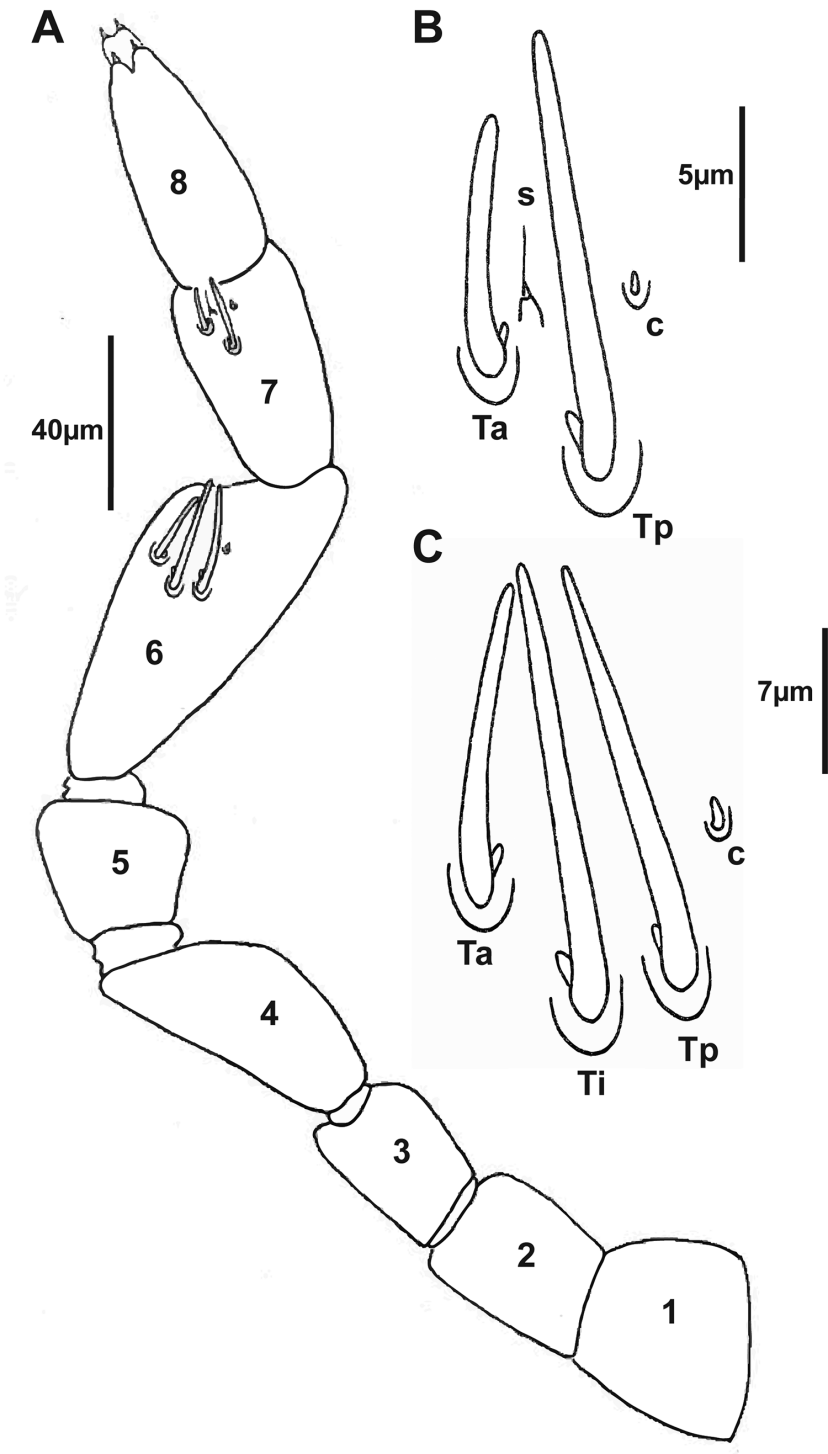
Antennal articles of the holotype *Lophoturus boondallus* sp. n. **A** Antenna with eight articles and the arrangement of sensilla on the 6^th^ and 7^th^ articles; articles 7^th^ and 8^th^ were equal in length; the arrangement of sensilla on the 6^th^ and 7^th^ antennal articles **B** Sensilla on the 7th antennal article; a conical sensillum (**c**), a long thick sensillum located posteriorly (**Tp**) and a short thick sensillum located anteriorly (**Ta**) with a setiform sensillum (**s**) located between these sensilla **C** Sensilla on the 6^th^ antennal article; a conical sensillum (**c**), a medium length thick sensillum (**Tp**), a long thick sensillum (**Ti**) and the short thick sensillum (**Ta**).

***Antennae*.** 8 articles (4 tiny, reduced sensory cones), 7^th^ and 8^th^ antennal articles are equal in length (Fig. 4A), which is characteristic of Lophoproctidae. The 6^th^ antennal article has 3 thick bacilliform sensilla (T) of differing lengths: medium sensillum posteriorly (Tp); the longest and thicker intermediate sensillum (Ti); a short sensillum anteriorly (Ta) with its socket located distally; and a conical sensillum posteriorly (c) (Figs 4C, 6F). The 7^th^ antennal article has 2 thick bacilliform sensilla (T), the anterior Ta shorter than Tp located posteriorly, with one setiform sensillum (s) between them plus a conical sensillum (c) located in the posterior position (Figs 4B, 6E). This pattern of sensilla on the 7^th^ article is common to all *Lophoturus* species.

***Clypeo-labrum***: Holotype has 10 setae, all half the width of the labrum. Setae on paratypes ranged from 10–12. Labrum surface setose, with tiny, backward facing hairs. Anterior margin of labrum with two whole lamellae, and a linguiform process present on each side of median cleft of labrum (Figs 3H, 7A).

***Gnathochilaria*.** Medial palps only, 58 sensilla on the palp of holotype (male) and 18–22 sensilla on paratypes (females) (Fig. 3F–G).

***Trunk*.** Comprised of 10 segments, 9 pleural projections, excluding the telson and caudal bundle; 13 pairs of legs. Collum – tergite 1 (smallest tergite) with trichome sockets arranged in 2 oval shapes laterally, connected by posterior curved rows of trichome sockets with a large gap in the middle. The collum is the only tergite with lateral protuberances bearing a small number of trichome sockets. In holotype, the collum has 52 (Left: L), 52 (Right: R) trichome sockets and the lateral protuberances with 7 trichome sockets on each side (Fig. 3B). Numbers varied in paratype females within a range of 46–58 trichome sockets in the collum and the number of lateral protuberances trichome sockets range 6–8. Tergites 2 to 10, have a pair of pleural projections located antero-laterally. The arrangement of tergal trichome sockets from tergites 2 to 10 typically have 2 latero-posterior oval groups with a few sockets extended on both ends with these groups separated by a large medial gap. Trichome sockets of tergite 2 in the holotype has 54 (L) and 54 (R) (Figs 3C, 6C), tergite 10 has 38 on both sides (Fig. 3D). In contrast, the trichome sockets of tergite 2 in paratypes ranged 54–66 and tergite 10 ranged 34–46 trichome sockets.

**Figure 5.**
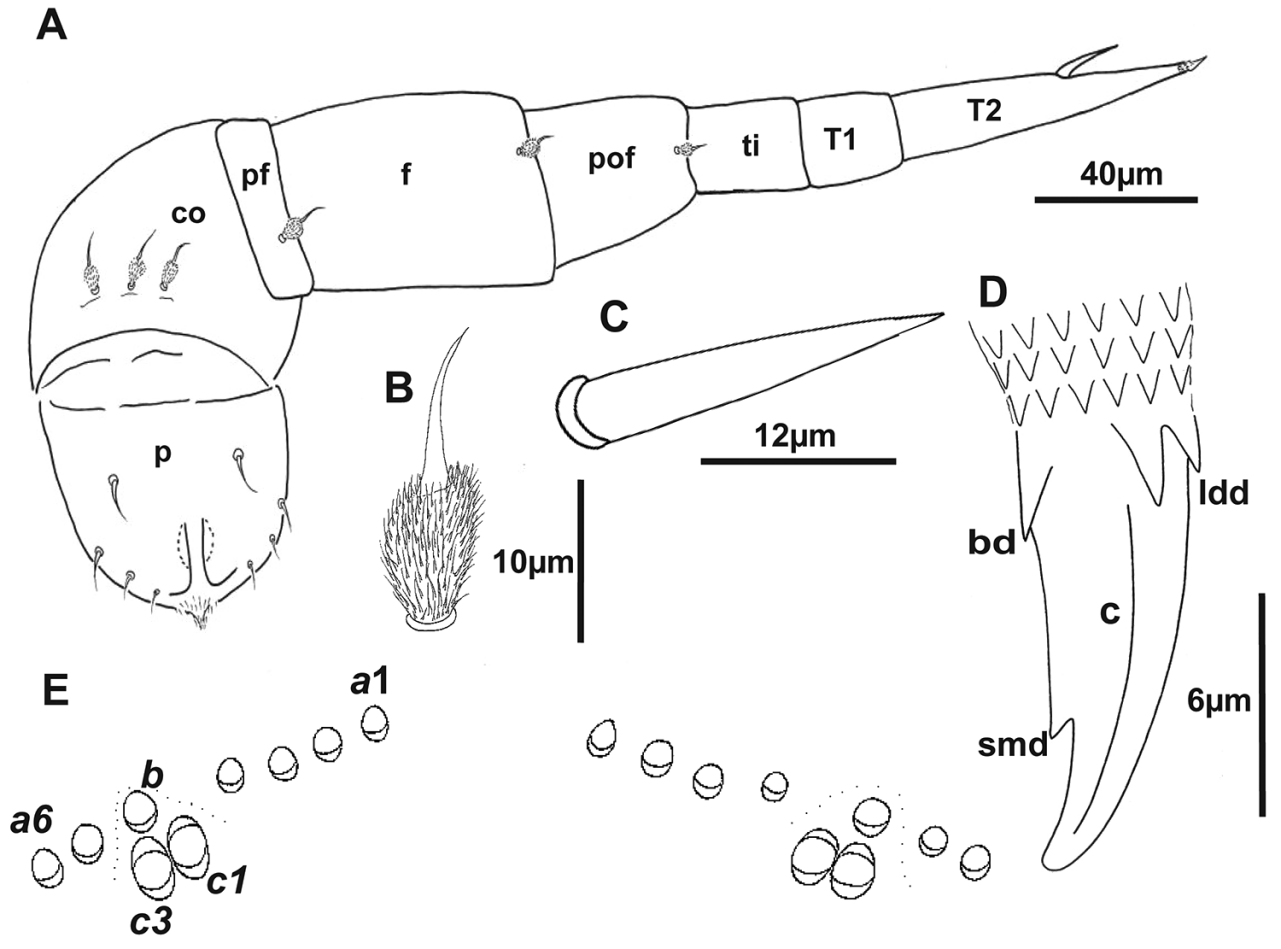
Holotype of *Lophoturus boondallus* sp. n. **A** The second left leg showing a penis (**p**), seven leg segments (**co** coxa, **pf** pre-femur, **f** femur, **pof**: post-femur, **ti** tibia, **T1** tarsus 1, **T2** tarsus 2 and a spine), a claw and its chaetotaxy (setae on the leg segments) **B** a pubescent oval seta **C** a spine on tarsus 2 **D** A simple claw structure with two latero-dorsal denticles (**ldd**), claw (**c**), a basal denticle (**bd**) and small denticle (**smd**) **E** The ornamental trichome sockets, located dorsally on the caudal bundle structure, with six trichomes ***a***, one trichome ***b*** and two trichomes ***c*** (***c***1 and ***c***3).

***Legs*.** Leg segments are named following Manton (1956). Legs 1 and 2 without trochanter, leg 1 also lacks tarsus 1. Chaetotaxy as follows: coxa 1: 2 pubescent oval setae, coxa 2: 3 pubescent oval setae, coxae 3–13: 0–4 pubescent oval setae; pre-femur, femur and post-femur with 1 pubescent oval seta (Fig. 5A–B), tarsus 2 with a spine (Fig. 5C). Posterior edge of last sternite has 0–4 pubescent oval setae similar to those present on the coxa and the number of these pubescent oval setae varies: 2 on the holotype and 0–4 on the paratypes. Sex organs in male: A pair of penes on the 2^nd^ coxa and coxal glands absent.

***Telotarsus*–*Claw*.** slender with two latero-dorsal denticles (ldd) equal in length, a basal denticle (bd) and a small denticle (smd) present near the tip of the claw (Figs 5D, 7B).

***Telson*.** Dorsal ornamental trichome sockets symmetrically arranged on each side, with 6 sockets of trichome ***a*** in the holotype; paratype females have 6–8 sockets of trichome ***a***, a single trichome ***b*** and two large protruding base sockets of trichome ***c***: ***c***1 and ***c***3 (The absence of ***c***2 is characteristic of lophoproctid species) (Fig. 5E).

***Caudal bundles*.** In the holotype male, the caudal bundle is formed by a single group of trichome sockets of uniform sizes; this structure is split ventrally with trichome socket-free tissue present and extending with a small gap dorsally toward the centre. 3 rows of the largest size barbate trichome sockets present, forming slightly uneven lateral rows that extend toward the centre of the caudal structure (Fig. 7C). In paratype females, the caudal bundle structure differed from the male, with two distinguishing structures apparent: the main dorsal structure, was similar to the male, and 2 latero-sternal structures with finer nest trichome sockets. These finer sockets located on the interior and were surrounded by 2 rows of caudal trichome sockets on exterior surface. A trichome socket-free area is present ventrally, extending with a small gap and connecting with central bare tissue with few barbate trichome sockets present. Caudal and nest trichome sockets are clearly separated. These structures gradually form a single caudal bundle structure (Fig. 7D). The structure of *L. boondallus* caudal bundles and their caudal trichomes is similar to those of *Monographis* (Polyxenidae) (Huynh and Veenstra 2013, 2015) and classified as Type II by Condé and Nguyen Duy-Jacquemin (2008).

**Figure 6.**
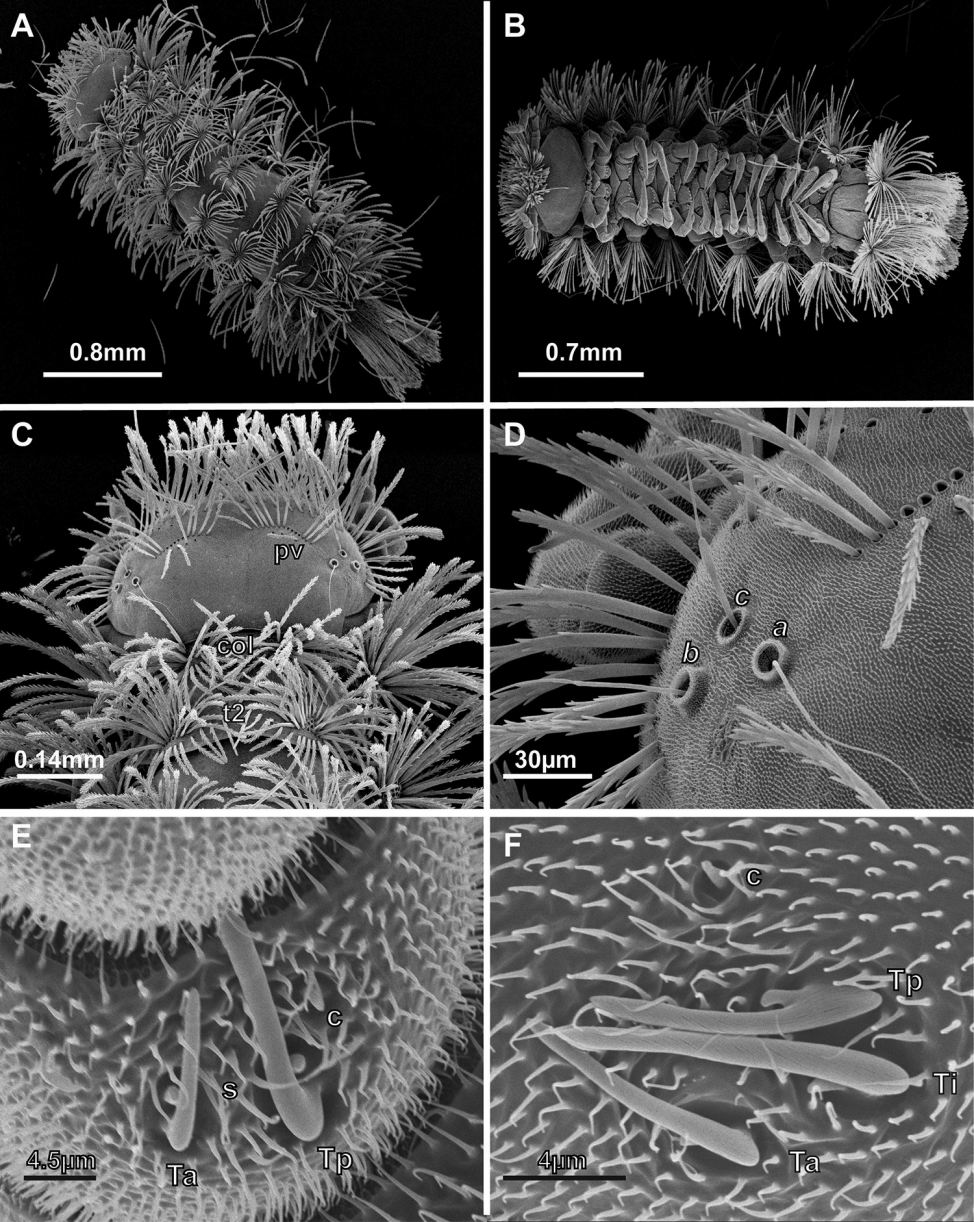
SEM (Scanning Electron Microscopy) images of *Lophoturus boondallus* sp. n. **A** A dorsal view of whole body showing the body trichome arrangements and the caudal bundle **B** A ventral view of whole body showed 13 pairs of legs **C** A head capsule displaying two posterior vertex trichome groups (**pv**), a collum (**col**) and tergite 2 (**t2**) **D** Trichobothria ***a*** (***a***), ***b*** (***b***) and ***c*** (***c***) showing different sizes in trichobothrium sockets **E** and **F** Antennal articles 6 and 7 with sensilla (**Ta**: thick sensillum located anteriorly, **Ti**: intermediated thick sensillum, **Tp**: posterior thick sensillum, setiform sensillum (**s**) and a conical sensillum (**c**)).

**Figure 7.**
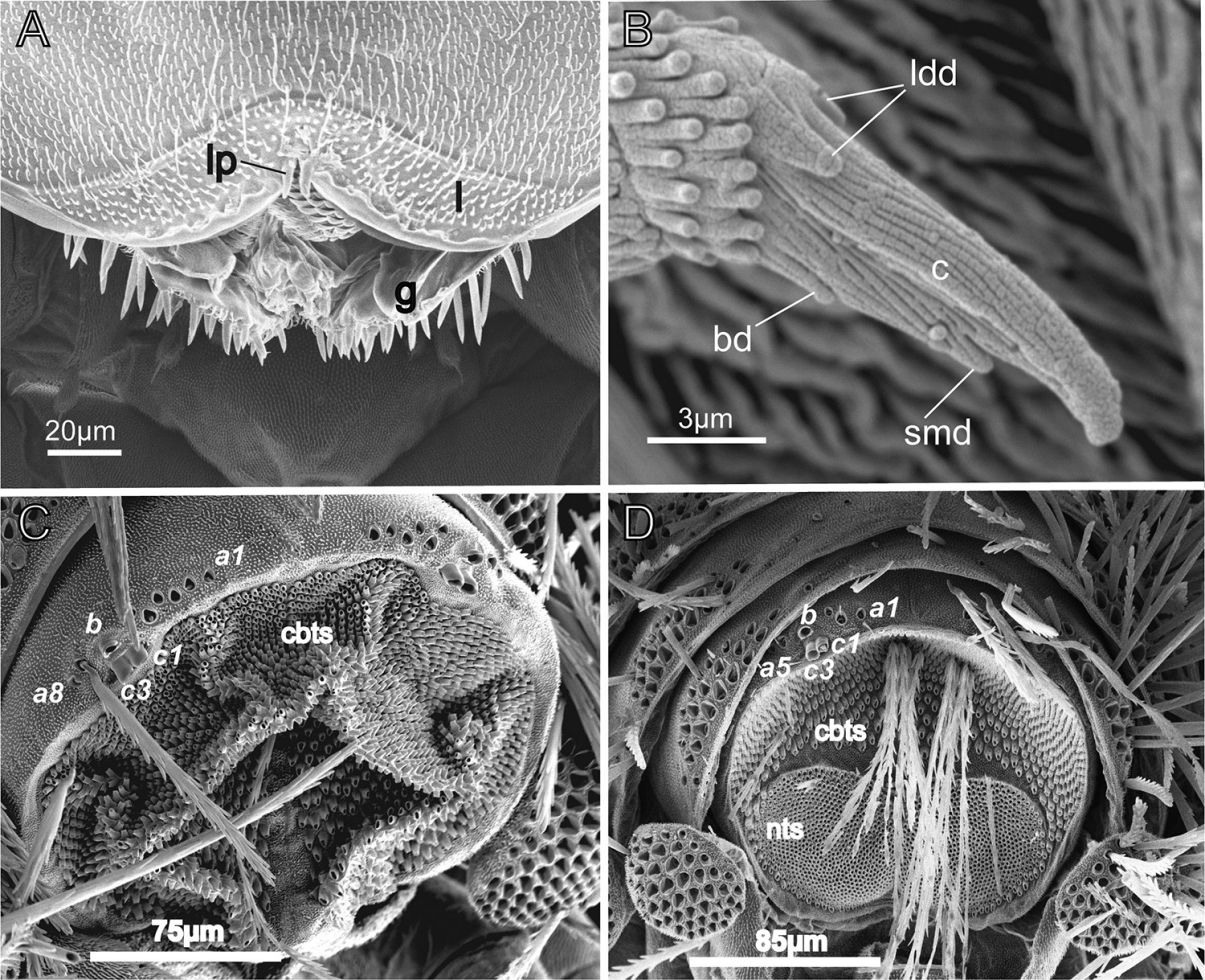
SEM images of *Lophoturus boondallus* sp. n. **A** Mouth part showed setose labrum (**l**) with typical two linguiform processes (**lp**) and sensilla from the gnathochilarium (**g**) **B** Simple claw with lateral dorsal denticles (**ldd**), claw (c), small denticle (**smd**) and basal denticle (**bd**) **C** Male caudal bundle showed an ornamental trichome sockets (***a***, ***b*** and ***c***) and the uniform caudal bundle trichome sockets (**cbts**); **D** Female caudal bundle structure displaying ornamental trichome sockets and two main parts: caudal bundle trichome sockets dorsally (**cbts**) and two nest trichome sockets (**nts**) ventrally.

###### Remarks.

*L. boondallus* differs from *L. queenslandicus* in being shorter in length, in having light orange colouration, a round arc-shaped body form when viewed in cross section. Furthermore, it also differs genetically from the other *Lophoturus* species. In contrast, *L. queenslandicus* is rusty brown with a yellowish light green median band dorsally and two darker brown strips laterally; body 2.4–2.8 mm long, with a flattened arc shape (Fig. 2).

##### Lophoturus molloyensis sp. n.

Taxon classificationAnimaliaPolyxenidaLophoproctidae

http://zoobank.org/79421543-DA49-454F-AA29-904170B24D9B

###### Holotype.

Adult male, stadium VIII – stage 13 leg pairs, was collected from Mount Molloy, Cairns region, Queensland; 16°41'10.50"S, 145°19'49.43"E, elevation 396 m, 8 December 2016.

###### Paratypes.

1 male, 4 females and one 12 leg pairs (subadult), collected with holotype.

###### Etymology.

*Lophoturus molloyensis* sp. n. is named after the collection location, Mount Molloy in the Cairns region of far north Queensland, Australia.

###### Diagnosis.

*L. molloyensis*, has 3 sensilla on the 6^th^ antennal article, and labrum has two linguiform processes. These features are typical of *Lophoturus*. In live, this species is white in colour with rounded body shape, covered with shorter trichomes. Body length of adults ranges from 1.4–1.8 mm, which distinguishes it from the longer *Lophoturus* species.

###### Description.

*Measurements*: Holotype male body length 1.4 mm; females (paratypes) (*n* = 4) range from 1.6–1.8 mm. Caudal bundle of male is slightly narrower in width and 0.2 mm in length than that of the female with 0.3 mm (Fig. 11A–B).

***Colouration*.** Head white and dark reddish brown in eye area; body yellowish-white with dull white pleural trichomes and bright white in caudal bundle (Fig. 2C).

**Figure 8.**
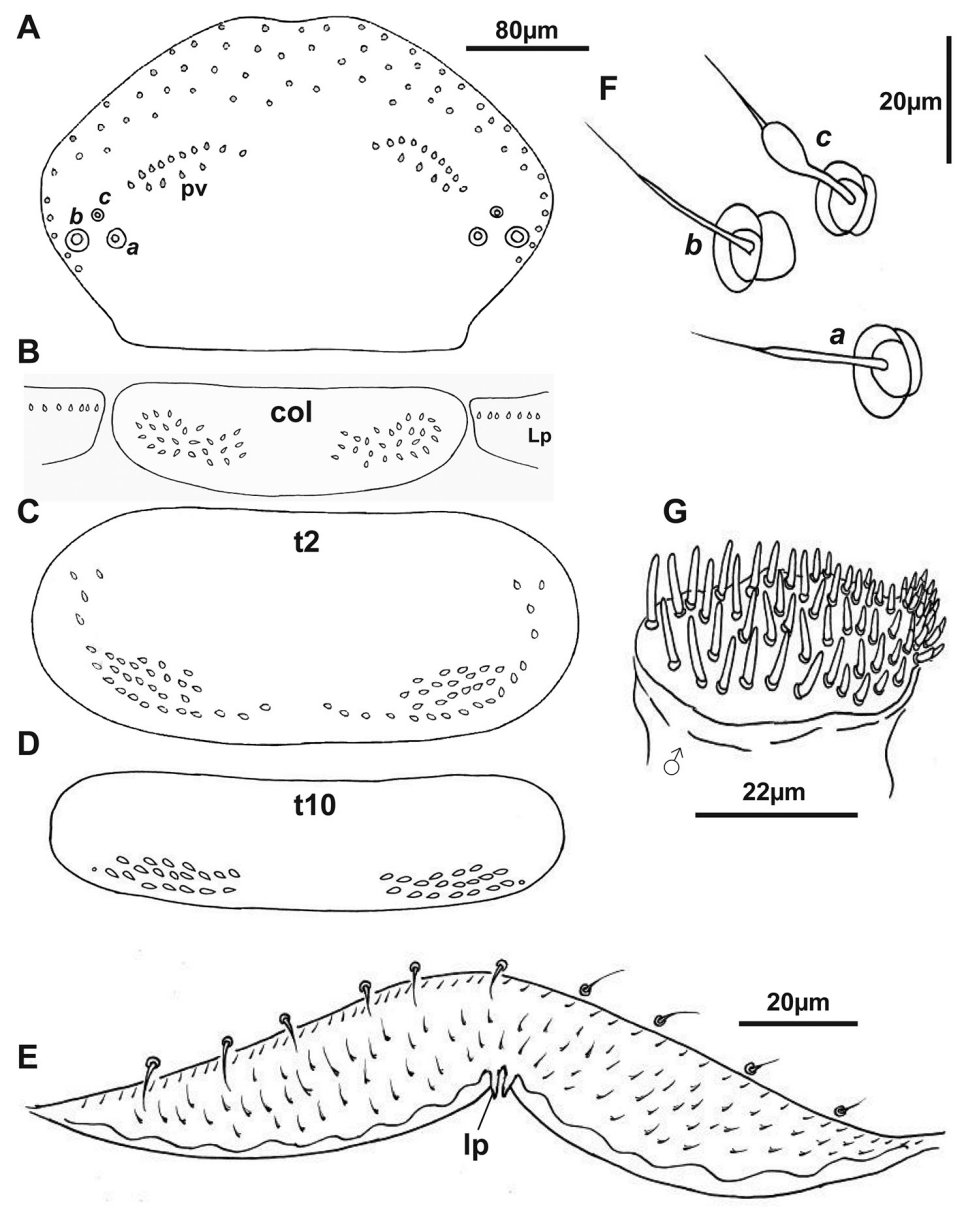
Holotype of *Lophoturus molloyensis* sp. n. **A** Head capsule, absence of ommatidia indicated, two posterior vertex trichome sockets (**pv**) and trichobothria ***a, b*** and ***c*** with the sockets only **B** the collum (**col**) and two lateral protuberances (**Lp**) **C** Tergite 2 (**t2**) and **D** The last tergite 10 (**t10**), showing the arrangement of trichome sockets **F** Trichobothria: ***a*** (the medium base socket located posteriorly) and ***b*** (the largest base socket located laterally) are typical thin sensory hairs, ***c*** with with a claviform funicle (the smallest base socket located anteriorly) **G** The male, right gnathochilarium showing numerous sensilla (ranged 56 – 58 sensilla in male) **E** Labrum showing a pair of linguiform processes (**lp**) and setose surface.

***Head*.** Ommatidia absent. Vertex with two posterior trichome groups, a large gap presents between them. Each group consists of 2 rows, the anterior, oblique row has similar sized trichomes. Posterior row has fewer trichome sockets with a narrow space between the anterior and posterior rows (Figs 8A, 11C). Holotype male has 10 + 10 trichome sockets in anterior rows and 4 + 4 trichome sockets in posterior rows; paratypes indicate that variation is common in this species, ranging from 9–11 (anterior rows) and 3–6 (posterior rows). Trichobothria: This species has the same structure and arrangement of trichobothria as seen all *Lophoturus* species: Trichobothria ***a*** and ***b*** are typically thin sensory hairs with narrow cylindrical funicles compared to trichobothrium ***c***, with a claviform funicle. (Figs 8F, 11D).

**Figure 9.**
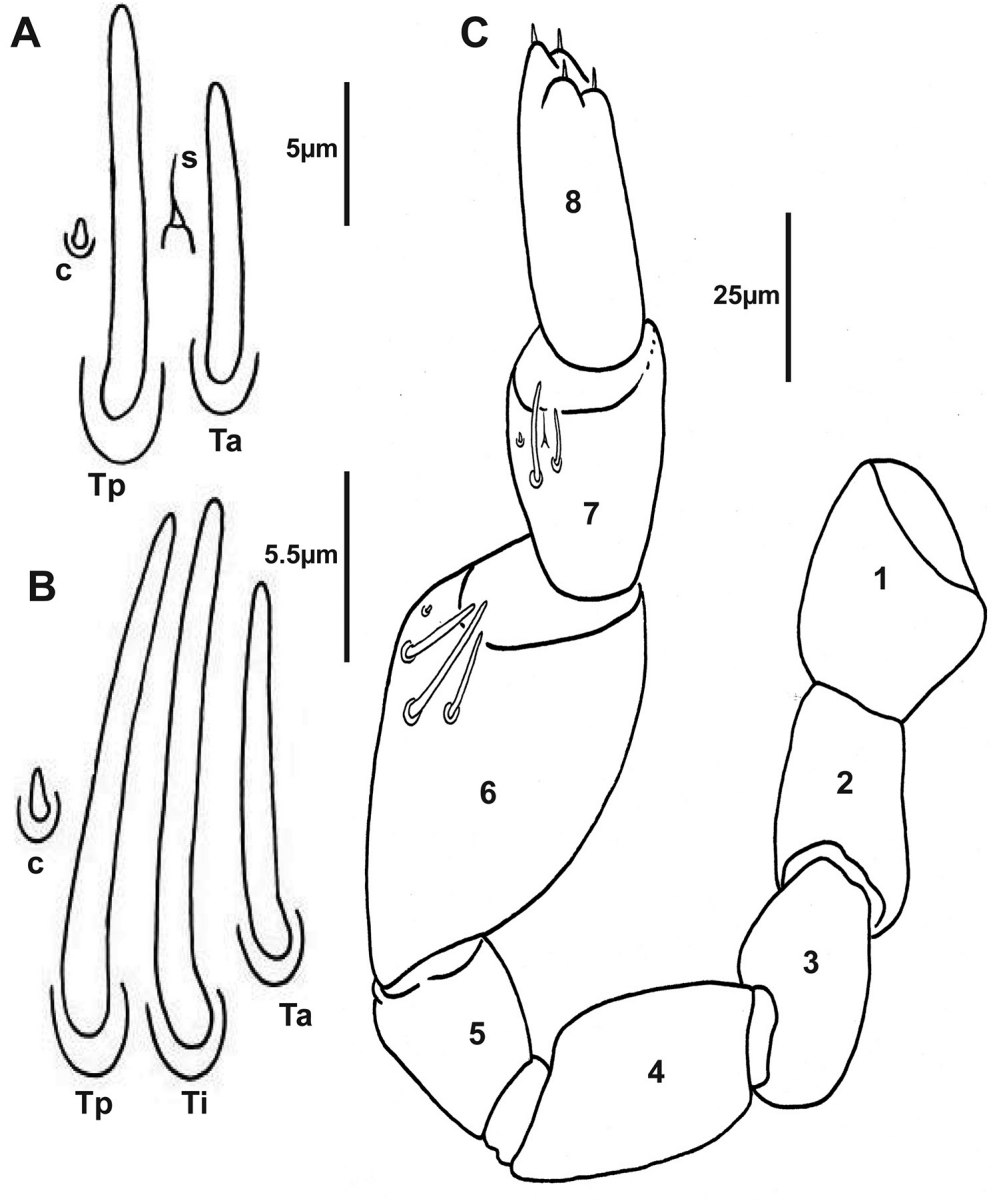
Holotype of *Lophoturus molloyensis* sp. n. **A** The arrangement of sensilla on the 7^th^ antennal article: A conical sensillum (**c**), a long thick sensillum located posteriorly (**Tp**) and a short thick sensillum located anteriorly (**Ta**) with a setiform sensillum (**s**) located between these sensilla **B** Sensilla on the 6^th^ antennal article: a conical sensillum (**c**), a medium length thick sensillum located posteriorly (**Tp**) and a long thick sensillum located intermediately (**Ti**) followed the short thick sensillum (**Ta**) **C** The left antenna with eight articles and the arrangement of sensilla on the 6^th^ and 7^th^ articles.

***Antennae*.** 8 articles, 7^th^ and 8^th^ antennal articles are equal in length (Fig. 9C). The 6^th^ antennal article has 3 thick bacilliform sensilla (T): Medium sensillum posteriorly (Tp), the longest, thicker intermediate sensillum (Ti), a short sensillum anteriorly (Ta) with its socket distal to other, and a conical sensillum posteriorly (c) (Figs 9B, 11E). The 7^th^ antennal article has 2 thick bacilliform sensilla (T), the anterior one (Ta) shorter than (Tp) located posteriorly, with one setiform sensillum (s) between them and a conical sensillum (c) in the posterior position (Figs 9A, 11E).

***Clypeo-labrum*.** Holotype has 10 setae, all shorter than half the width of the labrum. Setae on the paratypes ranged from 10–12. Labrum surface setose, with tiny, backward facing hairs. Anterior margin of labrum has two whole lamellae, and a linguiform process present on each side of median cleft of labrum (Figs 8E, 11F).

***Gnathochilaria*.** Medial palps only, 58 sensilla on the palp of holotype (male) and 18–22 sensilla on paratypes (females) (Fig. 8G).

***Trunk*.** Comprised of 10 segments, 9 pleural projections, excluding the telson and caudal bundle; 13 pairs of legs. Collum with trichome sockets arranged in 2 oval shapes laterally, connected by a posterior row of trichome sockets forming a line with a large gap in the middle. Lateral protuberances have a small number of trichome sockets. In holotype, the collum has 26 trichome sockets on both sides and the lateral protuberances have 7 trichome sockets on each side (Figs 8B, 11C). Numbers varied in paratype females within a range of 26–29 trichome sockets in the collum and the number of lateral protuberances trichome sockets range 4–6. All other tergites, from tergites 2 to 10, have a pair of pleural projections located antero-laterally. The arrangement of tergal trichome sockets from tergites 2 to 9 typically have 2 latero-posterior oval groups with a few sockets extending on both ends with these groups separated by a large gap. Trichome sockets of tergite 2 in the holotype have 33 on each side (Figs 8C, 11C), tergite 10 has two groups of 18 sockets both sides without any extended sockets, (Fig. 8D). In contrast, the trichome sockets of tergite 2 in paratypes ranged 30–34 and tergite 10 range was 16–19 trichome sockets.

**Figure 10.**
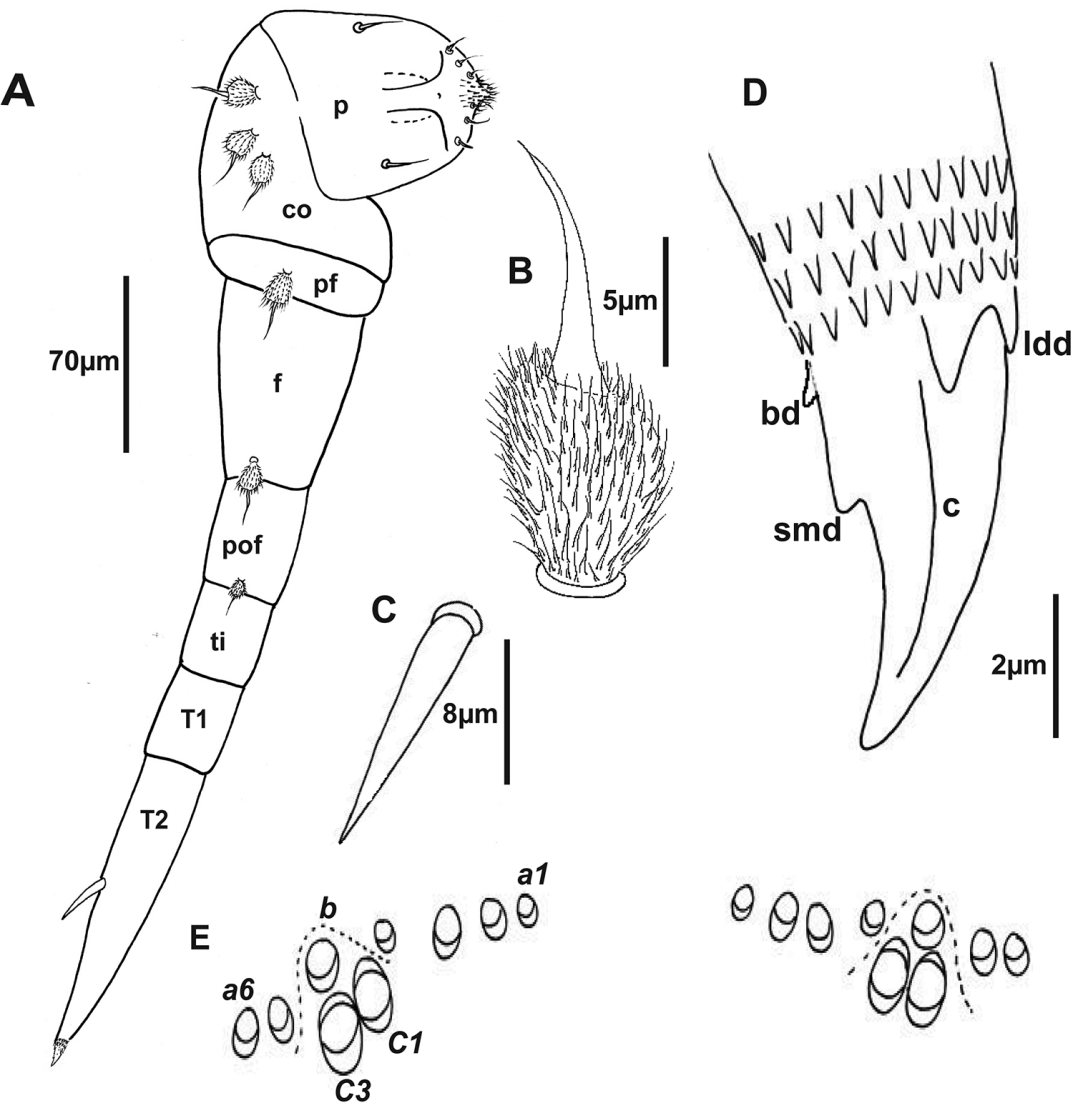
Holotype of *Lophoturus molloyensis* sp. n. **A** The second right leg with a penis (**p**), seven leg segments (**c** coxa, **pf** pre-femur, **f** femur, **pof** post-femur, **ti** tibia, **T1** tarsus 1, **T2** tarsus 2 and a spine), a claw and its chaetotaxy (setae on the leg segments) **B** A pubescent oval seta **C** A spine on tarsus 2 **D** A simple claw structure showing two latero-dorsal denticles (**ldd**), claw (**c**), a basal denticle (**bd**) and a small denticle (**smd**) **E** The ornamental trichome sockets, which located dorsally above the caudal bundle structure, with six trichomes ***a***, one trichome ***b*** and two trichomes ***c*** (***c***1 and ***c***3).

***Legs*.** Leg segments are named following Manton (1956). Legs 1 and 2 are without trochanter, leg 1 also lacks tarsus 1. Chaetotaxy as follows: coxa 1: 2 pubescent oval setae, coxa 2: 3 pubescent oval setae, coxae 3–13: 0–4 pubescent oval setae; pre-femur, femur and post-femur with 1 pubescent oval seta (Fig. 10A–B), tarsus 2 with a spine (Fig. 10C). Posterior edge of last sternite has 0–4 pubescent oval setae, similar those present on the coxa and the number of these pubescent oval setae varies: 4 on the holotype and 0–4 on the paratypes. Sex organs in male: A pair of penes on the 2^nd^ coxa and coxal glands absent.

***Telotarsus - Claw***: robust with two latero-dorsal denticles (ldd) equal length, a basal denticle (bd) and a small denticle (smd) present near the middle of the claw (Fig. 10D).

***Telson*.** Dorsal ornamental trichome sockets symmetrically arranged on each side, with 6 sockets of trichome ***a*** in the holotype; paratype females have 4–6 sockets of trichome ***a***, a single trichome ***b*** and two large protruding base sockets of trichome ***c***: ***c***1 and ***c***3 (Fig. 10E).

***Caudal bundles*.** These caudal structures similar in both sexes and like those of *L. boondallus* described above.

###### Remark.

*L. molloyensis* differs from both *L. queenslandicus* and *L. boondallus* in size, body length (about 1.6 mm), form and colouration. Body trichomes are short. The 12 leg pairs stage of this species may initially be confused with *L. madecassus* Marquet & Condé, 1950 as they have the same body length and appearance. Both have 8 pleural projections, but *L. molloyensis* has 12 leg pairs in subadult stage compared to *L. madecassus* which has 11 leg pairs in the adult stage.

**Figure 11.**
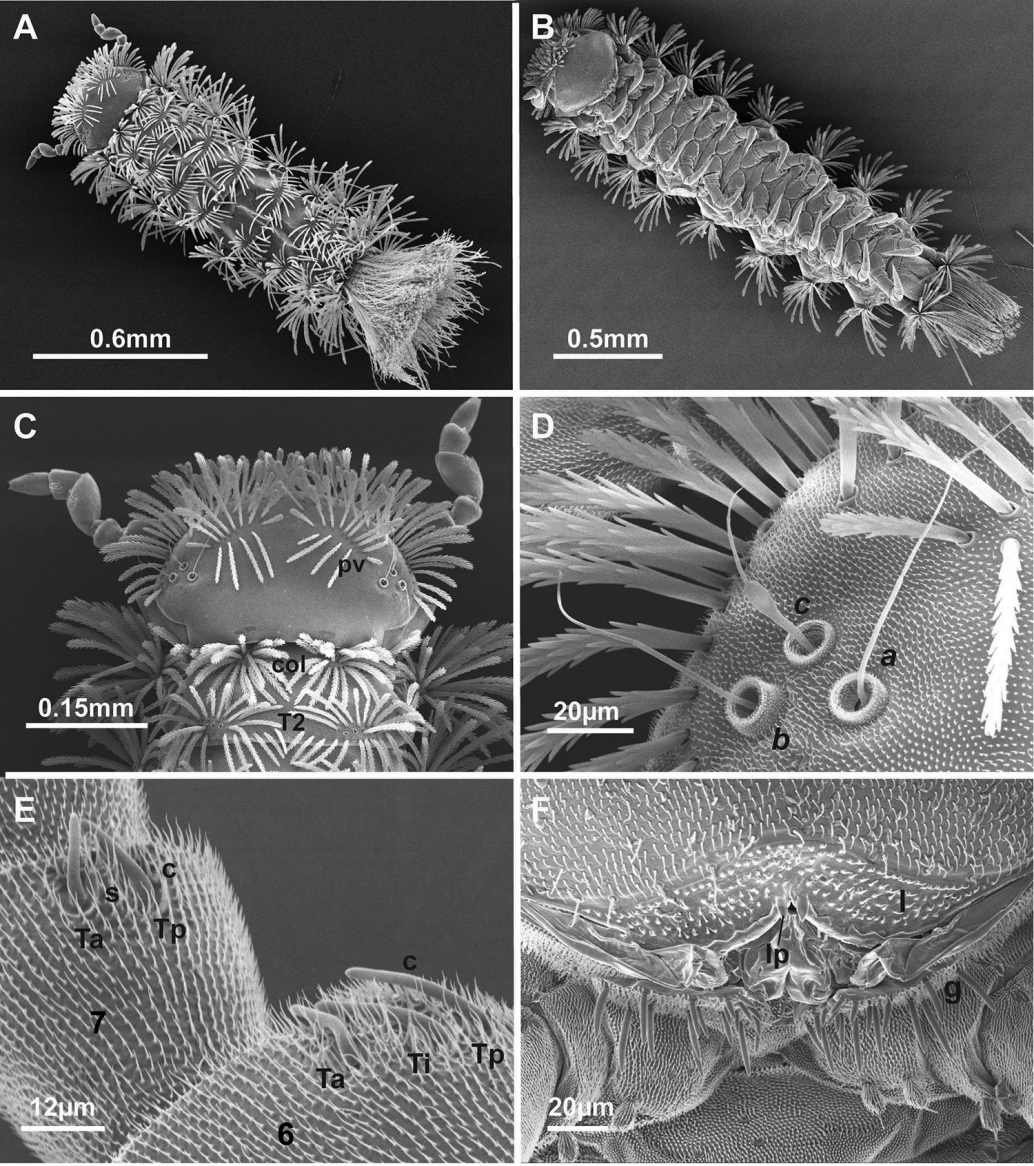
SEM images of *Lophoturus molloyensis* sp. n. **A** A dorsal view of whole body showing the body trichome arrangements and the caudal bundle **B** A ventral view of whole body showing 13 pairs of legs **C** A head capsule displaying two posterior vertex trichome groups (**pv**), a collum (**col**) and tergite 2 (**T2**) **D** The trichobothria: ***a, b*** and ***c***, showing different sizes in trichobothrium sockets **E** Antennal articles 6 and 7 with their sensilla (**Ta**: thick sensillum located anteriorly, **Ti**: intermediate thick sensillum, **Tp**: posterior thick sensillum, setiform sensillum (**s**) and a conical sensillum (**c**) **F** Mouth parts with setose labrum (**l**) with typical two linguiform processes (**lp**) and the gnathochilarium (**g**).

### Genetic analysis

**Figure 12.**
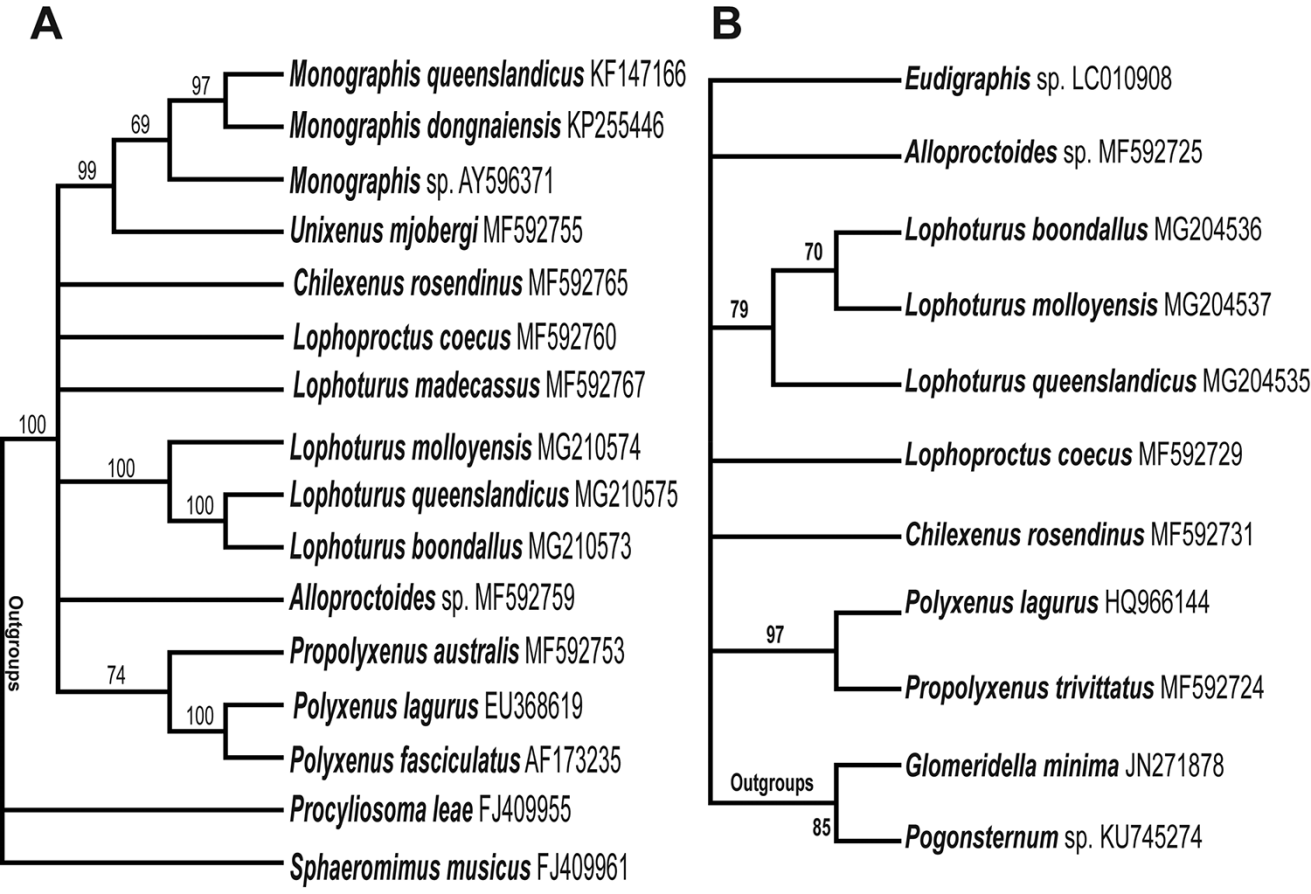
The molecular phylogenetic analysis by maximum likelihood method. **A** A consensus tree of sequences from 18S marker generated by the bootstrap test (1000 replications and the support values >50% shown on the nodes) yielded a strongly supported phylogenetic tree **B** The maximum likelihood test of sequences from COI marker provided the molecular phylogenetic tree of evolutionary history between each species. Again, this tree supported all *Lophoturus* species in the same clade as in the result of 18S.

The 18S maximum likelihood tree was generated by 1000 bootstrap replications yielded a strongly supported phylogenetic tree. The 18S region of the 3 sequences from studied *Lophoturus* species formed a statistically supported clade with all sequences of *Lophoturus* species. Phylogenetic analysis indicated that these species belong in the same genus *Lophoturus* (Fig. 12A).

Another bootstrap maximum likelihood tree based on comparison of the COI region of three *Lophoturus* species also yielded a strongly supported phylogenetic tree with the bootstrap value (>50%, shown on the nodes of the clade of three species) that these *Lophoturus* species are sufficiently distinct to warrant separation into 3 species (Fig. 12B).

Pairwise distances of the genomic DNA among *Lophoturus* species were analysed based on the maximum likelihood method to estimate of the evolutionary divergence between sequences. The genomic sequences based on the COI molecular marker showed significant percentage difference in the genetic distance between these *Lophoturus* spp.: *L. queenslandicus* was 16% genetic distance to *L. boondallus* sp. n. and 14% genetic distance to *L. molloyensis*. *L. boondallus* sp. n. and 14% genetic distance to *L. molloyensis* (Table 1).

**Table 1.** Pairwise distances of the sequences from three *Lophoturus* species were analysed based on the estimate of the evolutionary divergence between sequences from 18S and COI.

Species		Pairwise distance 18S		Pairwise distance COI	
*Lophoturus queenslandicus*	MG204535				
*Lophoturus boondallus*	MG204536	0.02		0.16	
*Lophoturus molloyensis*	MG204537	0.04	0.04	0.14	0.14

## Discussion

*L. queenslandicus* Verhoeff, 1924, *L. boondallus* sp. n. and *L. molloyensis* sp. n. all exhibit many similar morphological characteristics i.e. labrum with setose surface and a pair of linguiform processes, 3 thick sensilla (thick sensilla: located in anterior position (Ta), intermediate position (Ti) and posterior position (Tp)) on the 6^th^ antennal article, which are typical characteristics of genus *Lophoturus*. In preserved specimens where their colouration has gradually faded and trichomes damaged or lost, it is difficult to identify to species level. In live specimens, these species appear quite different based on body colouration and form: *L. queenslandicus* is rusty brown colour with a yellowish light green median band dorsally with two darker brown strips laterally; body length ranged 2.4–2.8 mm and it has a flattened arc body shape. *L. boondallus* is different in colour, being light orange with a rounded arc body shape, the adult body lengths often ranged from 1.6–2.2 mm. In contrast, *L. molloyensis* differs from the other two species having the shortest body length of about 1.6 mm and being white colour with round body shape (Fig. 2). However, there is an alternative way to identify these species, especially with the advances in molecular technology, by using genetic analysis. The molecular markers such as 18S and COI can help to confirm species identification.

## Supplementary Material

XML Treatment for Lophoturus

XML Treatment for Lophoturus queenslandicus

XML Treatment for Lophoturus boondallus

XML Treatment for Lophoturus molloyensis
